# Stretching to Reduce Pain-Related Disability Among Echocardiographic and Interventional Laboratory Employees—A Pilot Study

**DOI:** 10.1016/j.jscai.2024.101353

**Published:** 2024-05-02

**Authors:** Russell Gelfman, Brenden S. Ingraham, Gurpreet S. Sandhu, Amir Lerman, Bradley Lewis, Rajiv Gulati, Patricia A. Pellikka, Steven D. Higgins, Mandeep Singh

**Affiliations:** a Department of Physical Medicine and Rehabilitation, Mayo Clinic and Mayo Foundation, Rochester, Minnesota; b Department of Cardiovascular Diseases, Mayo Clinic and Mayo Foundation, Rochester, Minnesota; c Division of Clinical Trials and Biostatistics, Mayo Clinic and Mayo Foundation, Rochester, Minnesota

**Keywords:** health care workers, pain, stretches

## Abstract

**Background:**

Stretching improves range of motion and changes the viscoelastic properties of muscle-tendon units. We hypothesized that a regular stretching program would reduce the functional consequences of pain for employees working in echocardiographic, ultrasound, and interventional laboratories. This exploratory, proof-of-concept study was meant to inform expectations for future randomized, controlled studies.

**Methods:**

In this unblinded, nonrandomized, observational study, we enrolled 196 health care professionals working in the interventional and echocardiographic laboratories in the departments of cardiology and radiology at Mayo Clinic and Mayo Clinic Health System to perform 15-minute neck, upper extremity, low back, and lower extremity stretches for 1 year. The functional consequences of pain were self-reported by using the Disability of Arm, Shoulder, and Hand; Neck Disability Index; and Roland-Morris Questionnaire, which was administered at baseline and at 1 year to measure response to stretching. Monitoring with an assessment plan for injuries was undertaken. Employees who were pregnant, unable to do exercises, or under active orthopedic treatment, were excluded.

**Results:**

Of the 196 enrolled, 68 (35%) provided complete data at both baseline and follow-up. The majority of participants were over 40 years (n = 51; 72%) and female (n = 51; 72%). Participants performed stretches for 120.5 (IQR, 52-184) days over the year. The number of days of doing the stretches was well distributed across the study period with median quarters 1, 2, 3, and 4 of 32 (19-51), 32 (20-51), 31 (17-45), and 32.5 (12-47) days, respectively. The majority of participants (52.3%) stretched before, 18.9% stretched during and 28.8% stretched after work. Self-reported upper extremity disability improved in the treatment group with a significant decrease in the median Disability of Arm, Shoulder, and Hand score (5.2 to 2.6; *P* = .002). There was an absolute 4% decrease in the Neck Disability Index score, between baseline and 1-year follow-up (10% to 6%, *P* = .017). There was not a significant change in the Roland-Morris Questionnaire from baseline to follow-up (1 to 0; *P* = .287). No participant reported any stretch-related injuries.

**Conclusions:**

A routine stretching program may represent an attractive, low-cost, noninvasive option to reduce upper extremity musculoskeletal disability of employees working in the echocardiographic, ultrasound, and interventional laboratories. Larger randomized trials are needed to confirm the association.

## Introduction

The volume and complexity of fluoroscopically-guided interventional procedures have increased in the contemporary era.^1^ It has led to additional radiation exposure and has lengthened the time that personnel wear protective lead aprons. In 2007, approximately 1.1 million cardiac catheterizations and 622,000 percutaneous coronary interventions were performed in the United States.^2^ Volumes of structural interventions such as transcutaneous aortic valve replacements have now exceeded surgical aortic valve replacements.^3^ The number of percutaneous procedures performed worldwide is far greater when one includes transcutaneous mitral, pediatric, peripheral vascular interventions, ablations, and device implantations performed by electrophysiologists.^4^ The concomitant need for noninvasive imaging by echocardiogram, ultrasound, and other interventional procedures has also increased over time.^5^^,^^6^

Musculoskeletal pain is prevalent among health care workers and may be due to a combination of stress, overexertion, and awkward and prolonged postures. Repeated exposures and demands of activities can overload and exceed the threshold of tolerable stress resulting in musculoskeletal pain. In a previous multisite, case-control Mayo Clinic study that involved physicians and nonphysician allied health staff, 55% of personnel working in radiology and cardiology departments reported musculoskeletal pain, 30% sought medical care for pain, and 29% had pain at the time of the study.^7^ Despite being younger and having worked for fewer years, a higher number of technicians and nurses reported work-related musculoskeletal pain versus a control group. Addressing the high prevalence of musculoskeletal pain presents an opportunity to improve the work-related health of employees.

Regular stretching improves range of motion, reduces discomfort or pain, and improves the viscoelastic properties of muscle-tendon units. Therefore, it presents an attractive, low-cost, noninvasive option to improve musculoskeletal pain among employees working in a high-risk interventional environment.^8^^,^^9^ Prior studies are limited by lack of detailed description of stretches, shorter periods of intervention, and timing of stretches in relation to the work schedule.^8^^,^^9^ Assessing these variables might help to improve recommendations to optimize employees’ health. We hypothesized that such stretches performed for 1 year by personnel working in the radiology and cardiology departments, where they either wear lead aprons or use echocardiogram/ultrasound, at Mayo Clinics and Mayo Clinic Health System sites, would reduce their disability related to musculoskeletal pain involving the upper extremities, neck, and lower back.

## Participants and methods

Mayo Clinic consists of 3 major patient care facilities (Rochester, Minnesota; Scottsdale, Arizona; and Jacksonville, Florida), as well as Mayo Clinic Health System facilities in Minnesota (Mankato) and Wisconsin (La Crosse and Eau Claire), which also have interventional facilities. Employees working at these sites within the departments of cardiology and radiology were identified through Human Resources electronic databases. The Mayo Clinic Institutional Review Board approved the study, and medical records were not accessed as a part of this investigation.

### Survey tool

Personnel working in the above departments were invited to participate in filling out standardized questionnaires to assess their disability at baseline and at 1 year. With the assistance of the Mayo Clinic Survey Research Center, an electronic email survey was developed and sent to 4387 employees. Reminders were sent at 2, 7, and 14 days. In a nonrandomized fashion, all participants who agreed to do the stretching exercises were included in the active treatment group and participants who just filled out the survey and did not participate in the stretches were included as a comparison group. The survey was administered twice, first at baseline before the start of the stretching program and second at the conclusion of the study 12 months later. Reminders were sent once a week for 6 weeks at study completion. The survey generated self-reported baseline demographic information (age, sex, weight, and height) and baseline physical activities, like walking, bicycling, vigorous-intensity sports, fitness, or recreational (leisure) activities that cause a large increase in breathing or heart rate, like running or football, for at least 10 minutes continuously. Baseline work-related information (eg, exposure to procedures involving radiation, echocardiogram/ultrasound, use of protective equipment including the lead apron), and basic personal medical information related to employment in the echocardiographic, ultrasound, or an interventional laboratory, history of musculoskeletal pain, carpal tunnel syndrome, medical evaluation/treatment for musculoskeletal pain, cancer, cataracts or any medical condition. The survey is available in the Supplementary Material.

#### Validated questionnaires

The Disability of Arm, Shoulder, and Hand (DASH), Neck Disability Index (NDI), and Roland-Morris (RMQ) questionnaires were administered at baseline and at the conclusion of 1 year. For the DASH questionnaire, level of upper extremity musculoskeletal disability was determined using the 30-item DASH questionnaire.^10^, ^11^, ^12^ Its use has been validated in workers and applied in working populations.^13^, ^14^, ^15^ Normal values from the general population are available from the American Academy of Orthopedic Surgeons.^10^ The NDI is a validated instrument for assessing self-rated disability in people with neck pain. It has been used in both clinical and research settings.^16^ The RMQ is a validated questionnaire that has been used to assess physical disability due to low back pain in research and clinical practice, including workers.^17^^,^^18^

#### Intervention

**Figure 1:**
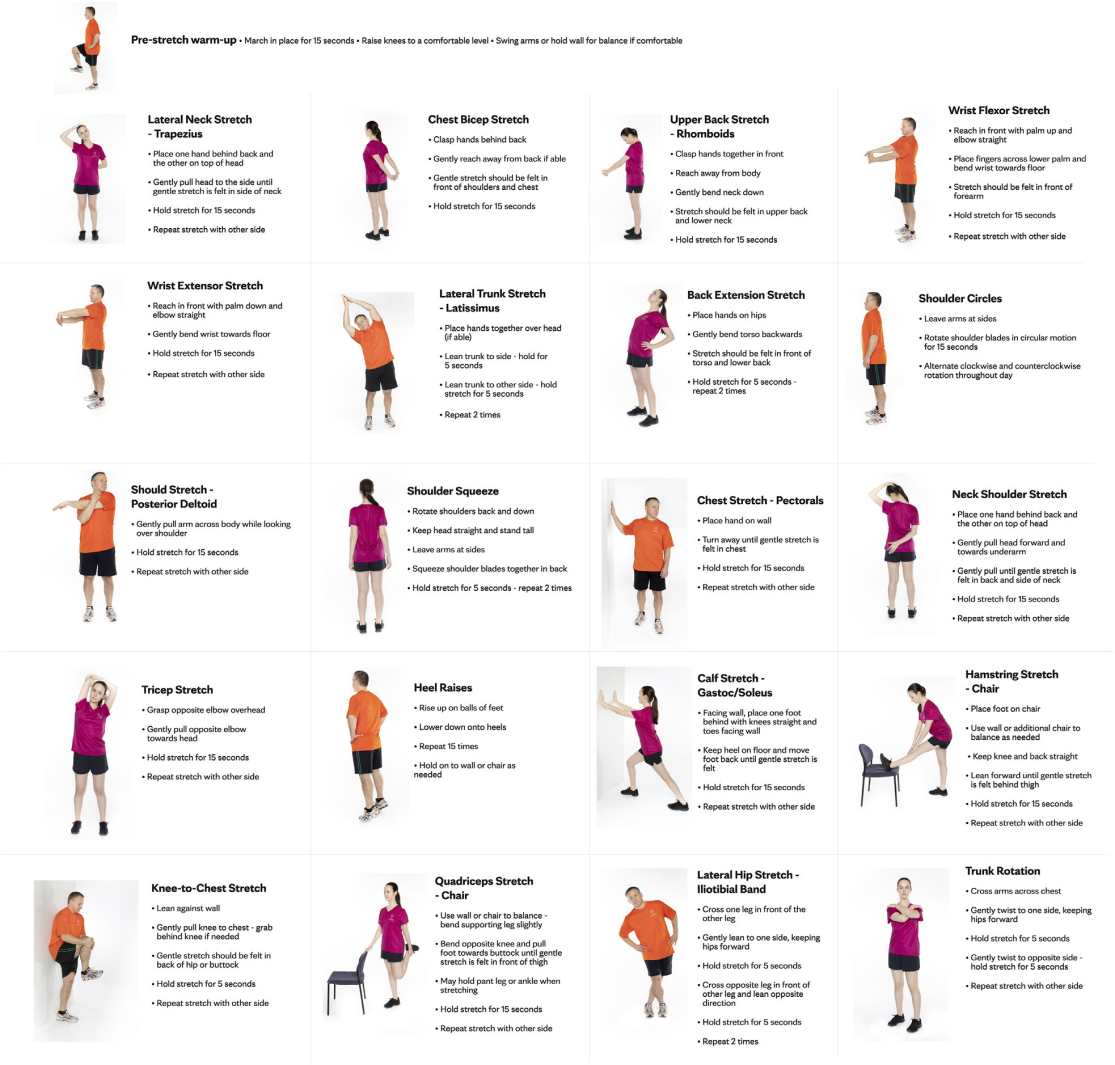
**Poster of exercises in the stretching program.**

This was a 1 year, unblinded, uncontrolled, and nonrandomized observational study. Participants in the active group were asked to perform 15 minutes of daily stretching exercises for 1 year targeting the neck, upper extremities, low back, and lower extremities. A physical therapist (PT) in the Department of Physical Medicine and Rehabilitation (S.D.H.), designed a 15-minute stretching routine that targeted the neck, upper extremity, low back, and lower extremity areas (Figure 1). These exercises were demonstrated in a video by a PT.^19^ Video demonstration was used to further engage and educate participants in the active treatment arm. Stretching could be done by the participants at any time of the day or night, at home or at work. Employees were asked to wear scrubs and soft sole shoes. The PT was available for guidance and for monitoring the progress. No attendance was recorded; however, we sent the participants a biweekly email/survey to note adherence to the stretching routine. No participant was removed from the intervention group.

#### Inclusion and exclusion criteria

Oral consent was required for inclusion in the study. Key exclusion criteria included pregnancy, inability to participate in the stretching exercises for any reason, preexisting musculoskeletal problems that required employees to seek active medical or orthopedic treatment.

#### Stretching-related injuries

Stretching has been demonstrated to be safe in work-related environments.^20^ Work-rest schedules and stretch routines were designed by the PT (S.D.H.). Careful assessment, monitoring, and review of stretch-related injuries were done and reviewed from the biweekly questionnaire. We designed a program of controlled static and active stretching exercises (self-stretching), which generally involves moving a limb through its full range of motion to the end ranges for a comfortable hold. This is less likely to lead to stretch-related injuries compared to ballistic stretching, which includes rapid, alternating movements or “bouncing” at end range of motion. Due to the increased risk of injury, ballistic stretching is not typically recommended.

### Statistical analysis and power considerations

Continuous variables are presented as median (IQR) and discrete variables as count (percentage). Group comparisons were tested using Kruskal–Wallis for continuous variables and χ^2^ or Fisher exact test for categorical. Participants were excluded from analysis if they did not fill in both a baseline and follow-up questionnaire. Although this is an observational study involving those who performed the exercises for 1 year, those who did fill out both the baseline and follow-up forms but did not perform the stretches were considered controls (n = 4) and used as comparators for the active participants (n = 64). The change in DASH, NDI, and RMQ scores between groups was tested using a Kruskal–Wallis test, and differences between baseline and follow-up were tested using the Wilcoxon signed-rank test. RMQ score was split based on those who start with lower back pain (RMQ score >0) and those who do not (RMQ score = 0). The number of participants developing some level of lower back pain (RMQ score >0) was tested using the McNemar test. The change in RMQ score for those starting with lower back pain was again tested using the Wilcoxon signed-rank test.

## Results

### Survey response and study population

We received baseline survey responses from 479 clinical employees within the departments of cardiology and radiology among the 6 Mayo Clinic patient care facilities. Of these, 196 agreed to participate in the study, which began 59 days from the time the first survey was sent. Of those who enrolled, 68 (35%) had completed data at both baseline and follow-up (n = 26 cardiology, n = 42 radiology). Fifty (72%) respondents were over 40 years of age, with 72% female. There were 53 (74.6%) respondents who reported being involved with procedures that involve radiation. The most commonly defined job description was technician/technologist (16 [22.5%]) followed by nurse (9 [12.7%]), physician (11 [15.5%]), and other (eg, manager, supervisor, medical assistant) (35 [49.3%]) with median experience (years) being 16.5, 18.5, 20.0, and 15.0, respectively, in their current positions (Table 1).

**Table 2:** Participant demographics.

	No radiation	Radiation	*P* value
Age			.15
<20 y	0 (0.0%)	0 (0.0%)	
20-30 y	4 (22.2%)	2 (3.8%)	
31-40 y	3 (16.7%)	12 (22.6%)	
41-50 y	4 (22.2%)	15 (28.3%)	
51-60 y	6 (33.3%)	23 (43.4%)	
61-70 y	1 (5.6%)	1 (1.9%)	
>70 y	0 (0.0%)	0 (0.0%)	
Female sex	16 (88.9%)	35 (66.0%)	.06
Body mass index, kg/m^2^	23.5 (21.7-29.5)	26.6 (23.5-30.0)	.26
Years in current profession	15.0 (4.5-21.8)	18.5 (7.8-27.2)	.26
Prior musculoskeletal condition	4 (22.2%)	9 (17.0%)	.62

Values are n (%) or median (IQR).

### Baseline characteristics

The baseline demographic characteristics of our study population are summarized in Table 2. Employees with occupational exposure to procedures involving radiation were more likely to be men (34% vs 11%; *P* = .06) and work in radiology (47.2% vs 33.3%; *P* < .001). There was no difference in age, body mass index, years in the current profession, or pre-existing musculoskeletal conditions among employees with occupational exposure to procedures involving radiation versus those not involved in such procedures. Self-reports of walking, bicycling, vigorous-intensity sports, fitness, or recreational (leisure) activities that cause a large increase in breathing or heart rate, like running or football, for at least 10 minutes were captured.

**Table 1:** Job description.

	Control	Treatment	Total	*P* value
Job description				.62
Physician or APP	0	11	11	
Nurse	0	9	9	
Technologist	1	15	16	
Other	3	32	35	

APP, advanced practice provider.

There were 118 (60.8%) participants who reported walking or using a bicycle for at least 10 minutes continuously to get to places for a median of 5 (4-5) days per week. 112 (61.9%) participants reported doing moderate-intensity sports, fitness, or recreational (leisure) activities that cause a small increase in breathing or heart rate such as brisk walking, cycling, swimming, or playing volleyball for at least 10 minutes continuously for a median of 3 (2-5) days per week; and 79 (41.1%) participants reported doing vigorous-intensity sports, fitness, or recreational (leisure) activities that cause a large increase in breathing or heart rate like running or football for at least 10 minutes continuously for a median of 3 (3-5) days per week. There were 30 (15.3%) who answered “no” to all the activity questions.

### Stretching exercises

The stretching exercises targeted both upper and lower extremities, low back, and neck areas (Figure 1). The stretching exercises could be completed within 15 minutes. Patients had a median of 120.5 (52.0-184.0) stretching days, well distributed across the study period with quarters 1, 2, 3, and 4 having median stretching days of 32.0, 32.0, 31.0, and 32.5, respectively. Most participants preferred stretching before work (52.3%), whereas 18.9% preferred stretching during work, and 28.8% preferred stretching after work. Adherence to the stretching protocol was monitored by biweekly questionnaires. Sixty-seven employees regularly returned the questionnaire that asked 2 questions on how many days the employee exercised in the previous 2 weeks and for how many days they performed stretches.

### DASH, NDI, and RMQ questionnaires

**Figure 2:**
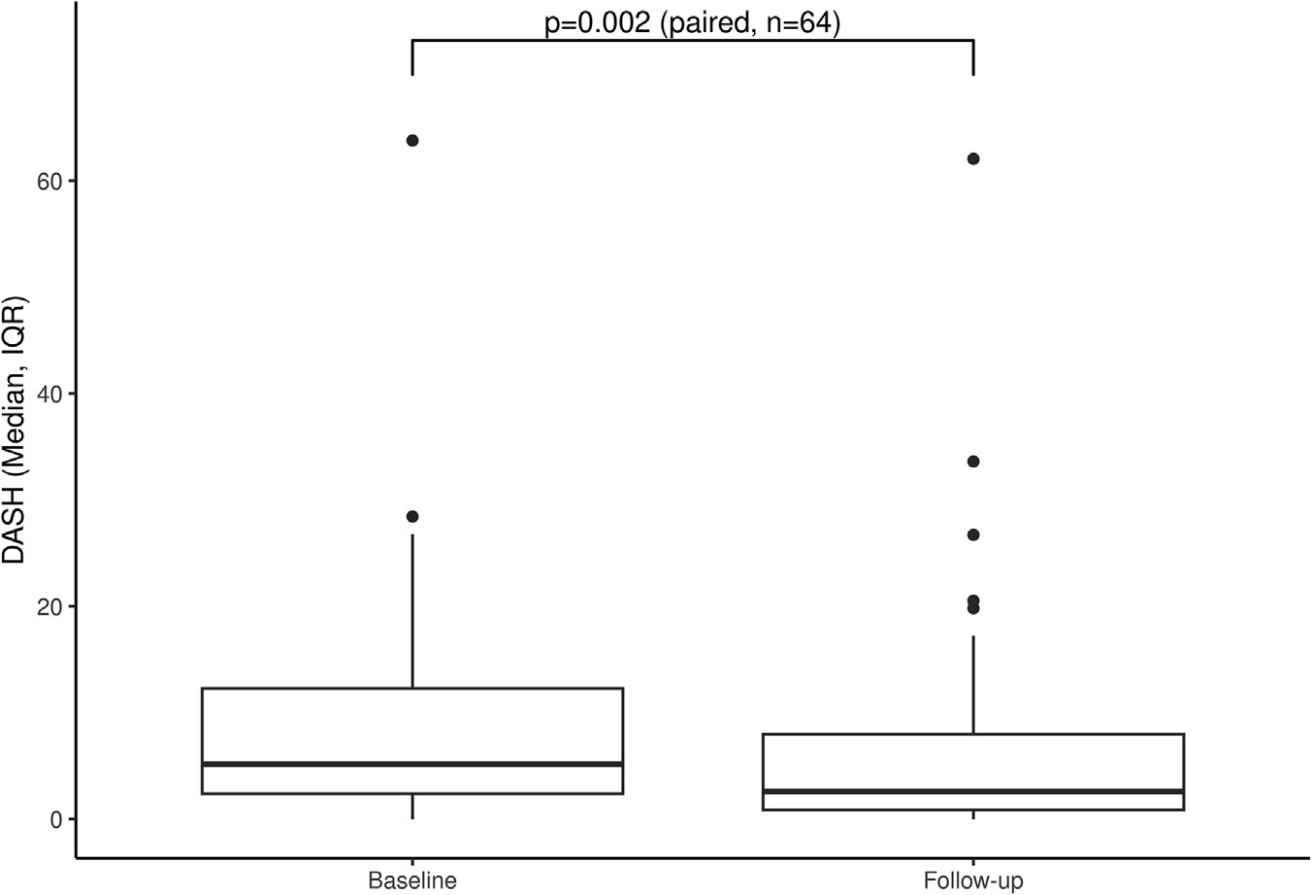
**Boxplot of Disability of Arm, Shoulder, and Hand (DASH) comparing baseline to follow-up for the treatment group.** Three participants were missing their follow-up DASH and have been excluded.

**Figure 3:**
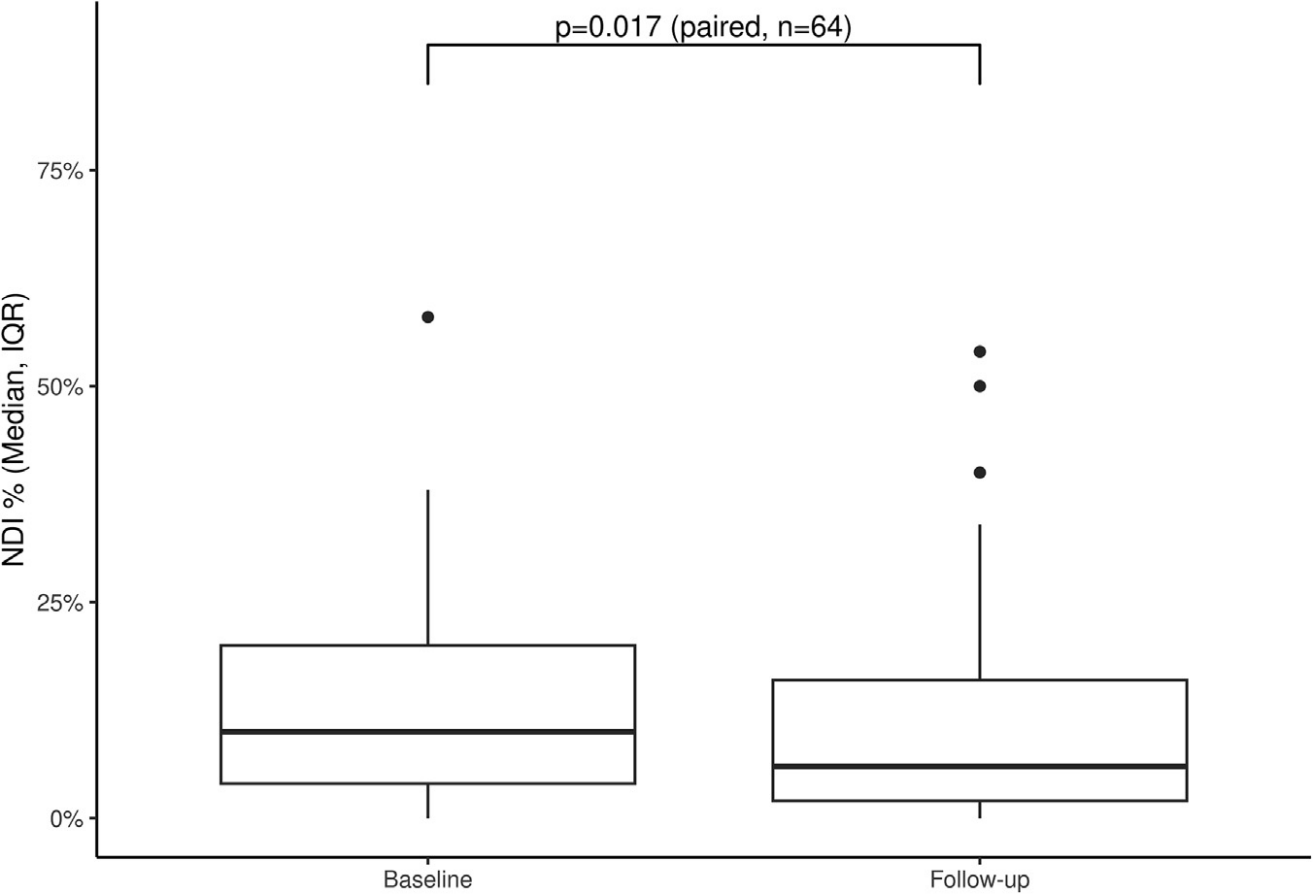
**Boxplot of Neck Disability Index (NDI) comparing baseline to follow-up for the treatment group.**

**Figure 4:**
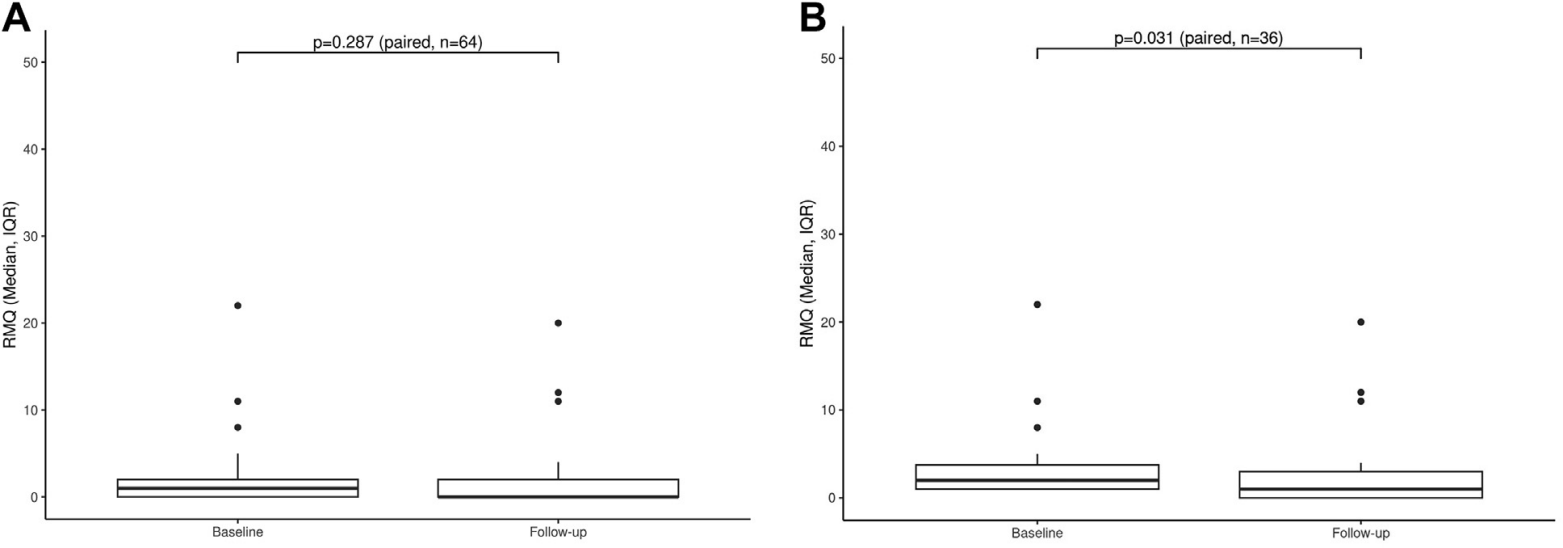
(**A**) Box plot of Roland-Morris Questionnaire (RMQ) comparing baseline to follow-up for the treatment group. (**B**) Box plot of RMQ at baseline and follow-up of participants with musculoskeletal pain at baseline (RMQ >0).

DASH questionnaire targeted the shoulder, arm, and hand areas whereas NDI focused on the neck and RMQ on the lower back. There was a significant decrease in the level of upper extremity disability as reflected in the DASH questionnaire between follow-up (2.6; IQR, 0.9-8.0) and baseline (5.2; IQR, 2.2-12.1) (Wilcoxon signed-rank test *P* = .002) (Figure 2). Neck disability, as assessed utilizing the NDI questionnaire, showed improvement (1 year follow-up [6.0%; IQR, 2.0%-16.0%] and baseline [10.0%; IQR, 4%-20%] [Wilcoxon signed-rank test *P* = .017]), (Figure 3). There was not a significant change in the RMQ from baseline (1; IQR, 0-2) to follow-up (0; IQR, 0-2) (Wilcoxon signed-rank test *P* = .287) (Figure 4A). Twenty-nine participants started with RMQ scores of 0 indicating no lower back pain. Of these 29 participants, 5 went on to develop some level of lower back pain (McNemar test *P* < .001). Of the remaining 36 who started the study with lower back pain, a significant reduction in back pain was observed from baseline (2; IQR, 1-4) and follow-up (1; IQR, 0-3) (Wilcoxon signed-rank test *P* = .031) (Figure 4B).

In the comparison (control) group, there was an increase in DASH score compared to the exercise (intervention) group, which saw a decline (11.2 [5.6-11.6] vs –1.2 [–4.3 to 0.0]; *P* = .03), though the control group size was admittedly small. There was no significant difference in the change in the NDI score between the control and the treatment group (–3 [–13 to 2] vs –2 [–8 to 2]; *P* = .63). There was no significant difference in the change in the RMQ score between control and intervention groups (0 [–0.5 to 1.3] vs 0 [–1 to 0]; *P* = .58). Importantly, no participant withdrew or reported stretch-related injuries.

## Discussion

Here we report a prospective, no-cost, minimal-risk, and potentially effective intervention to reduce musculoskeletal discomfort that did not require any special equipment or monitoring. The stretches could be performed any time of the day at the convenience of the health care employees working in the catheterization, echocardiographic, or radiology laboratories. This study was a multicenter observational study involving employees exposed to the occupational hazards of participating in procedures involving radiation and echocardiography. It includes attending physicians, technicians, nursing staff, and trainees. Our study intervention was designed by a PT who instructed the intervention in detail to study participants, included a realistic timeframe of the 1-year intervention phase, and used validated questionnaires to assess and compare self-reported disability at the hand/shoulder, neck, and lower back areas. There was an effort to document that stretches were done regularly with periodic monitoring for assessing compliance.^8^ There were no injuries reported.

Employees in a prior study from our center, who were involved in procedures that used radiation, reported a 67% prevalence of musculoskeletal pain, were more likely to seek medical care for their pain, and were more likely to have had an ergonomic evaluation.^7^ Female sex and increased time spent in the interventional lab wearing the lead apron were identified as major risk factors for reporting a higher prevalence of pain. The cause of the pain in the nonphysician interventional lab staff was not identified in that study, but it may relate to an increased exposure to physical stresses. Unlike their physician counterparts who regularly rotate out of the interventional laboratory, technicians and nursing staff typically do not. An additional factor may be that technicians and nursing staff are exposed to different physical stressors than physicians. Involvement in patient transfers on and off the interventional table and applying compression after sheath removal are both examples of typical nonphysician, work-related duties that can negatively affect the musculoskeletal system. Similarly, most echocardiographic images are obtained by the echocardiography technologists. We previously reported that cardiac sonographers reported work-related musculoskeletal pain more frequently than a control group (88% vs 40%; *P* < .001), sought medical care for their work-related pain more often (55% vs 21%; *P* < .001), and missed more work due to pain (35% vs 12%; *P* < .001).^21^

A higher prevalence of musculoskeletal pain among interventional cardiologists has been previously demonstrated.^7^ Forty-two percent of interventional cardiologists reported a history of musculoskeletal pain and higher prevalence was noted with higher case volumes and more years in practice. Despite the formation of working groups to reduce occupational risk and improve ergonomic efficiencies, little progress is noted in efforts to reduce ergonomic risk and study interventions that improve musculoskeletal pain in this high-risk group.

In this study, stretching improved self-reported upper extremity disability in the treatment group with a significant decrease in the median DASH score. We observed significant improvement in the DASH score based upon the observation that in addition to a reduction in the active participants, there was also a worsening of the score in the controls, underscoring the potential benefit of regular stretches on upper extremity disability. A higher proportion of participants did not have low back pain at baseline; however, a significant reduction in back pain was observed on follow-up from baseline (1 from 2; *P* = .031) among participants who complained of low back pain at baseline (Central Illustration).

**Central Illustration:**
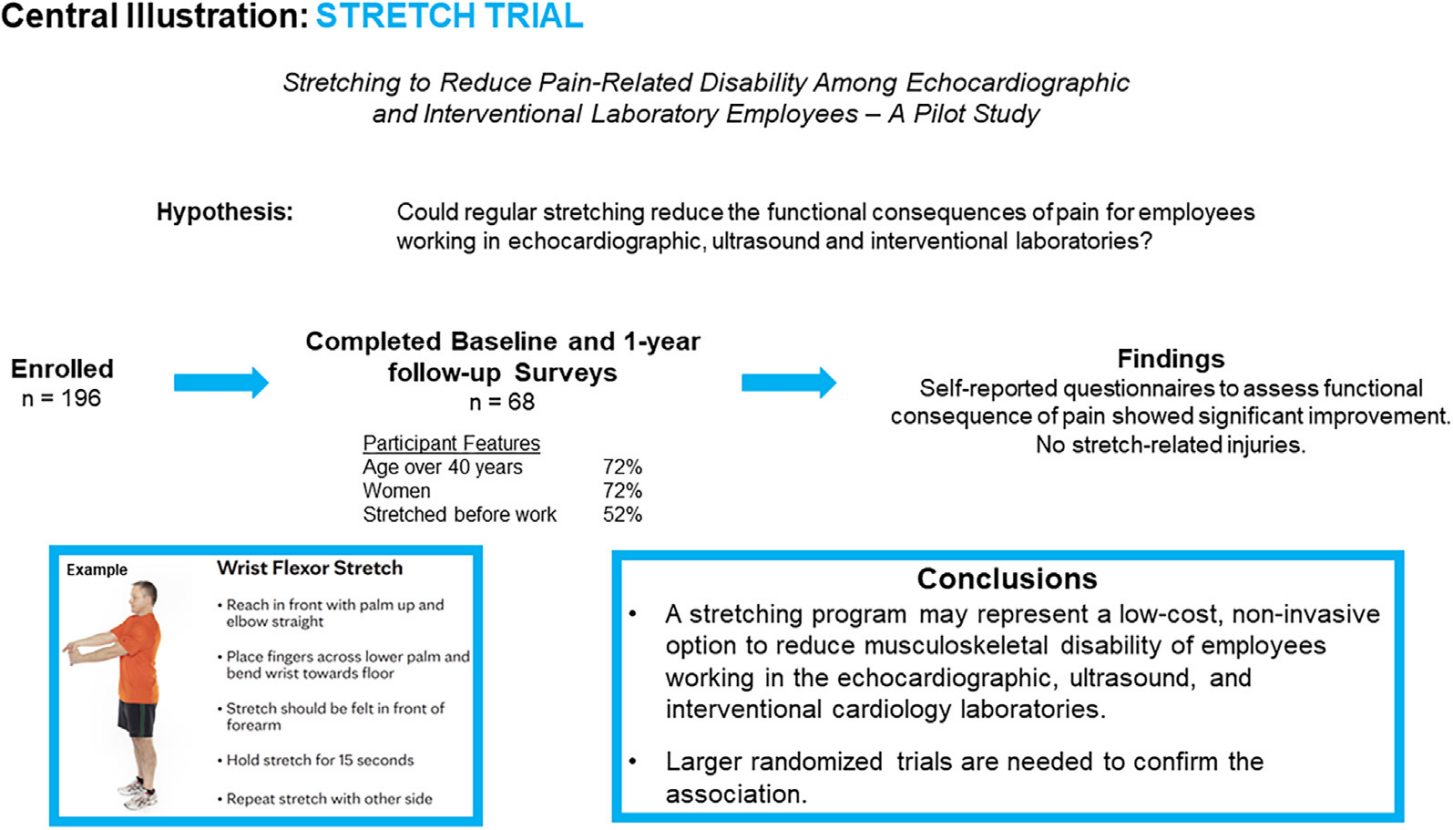
Regular stretching exercises reduce pain in echocardiographic, ultrasound, and interventional laboratory employees. Stretching is a low-cost, non-invasive way to reduce musculoskeletal disability.

Stretching exercises have demonstrated reduction in musculoskeletal pain.^20^^,^^22^, ^23^, ^24^ The established physiologic benefits of stretching are realized through purported gain in range of motion, favorable viscoelastic changes at the tendon-muscle units, and harmonizing muscle contraction with the peak force.^9^ However, the benefit of the stretches is less well-studied in relation to occupation (health care workers), timing of the shift (before, during, or after), or type of stretches (static, proprioceptive neuromuscular facilitation or ballistic). In addition, there is no unanimity about the current programs that involve stretches with regard to their effectiveness, need for supervision, frequency, and duration.^25^ For this trial, we used controlled static and active stretching (self-stretching) exercises, designed by a PT who works in our Work Rehabilitation Center, for the needs of personnel working in the catheterization, echocardiographic, and ultrasound laboratories.

Although beneficial, in addition to interventions like stretching, efforts will likely need to be directed toward reducing physical stresses among the employees working in cardiac catheterization, echocardiographic, and ultrasound laboratories.^24^ Efforts at limiting procedure time, regular ergonomic evaluations with associated training, and periodic rotation out of the respective lab suites may lessen musculoskeletal pain. Improving efforts in monitoring and implementing ergonomics including integration of lighter or nonlead-based radioprotective material may reduce occupational stresses. Robotic interventional with remote monitoring capabilities will obviate the need to wear lead aprons, reduce the number of personnel in the laboratories that require radiation, and also reduce the proximity of the operator from the radiation source.^26^

### Limitations

Despite the positive findings of the observed intervention, a significant limitation of our study is the absence of randomization or concealment of allocation. The nonrandomized, noncontrolled, unblinded structure means that it is possible that unmeasured variables (eg, baseline stretching habits) influenced some findings. Another issue of concern is the low rate of participation. This is not entirely surprising as the level of participation in health promotion programs is typically low.^27^ The results will need to be validated in a larger, randomized trial, perhaps with the addition of incentives for active exercise participants and controls. There is the possibility that employees without work-related pain or those not exposed to radiation might have felt less motivated to participate, leading to a response bias. In addition, employees who developed pre-existing musculoskeletal pain before the initiation of our study might have stopped working in the interventional laboratory and moved to noninterventional jobs in the same department. Although uncommon, it would have biased the results in favor of the null. More than 50% of the employees did stretching exercises before their shift; the effect of timing of these exercises to the shift could not be answered from our study. Even as we sent biweekly surveys to monitor progress, injuries, and compliance, the stretches were not monitored.

## Conclusion

Regular stretches may represent an attractive, safe, low-cost, intervention to reduce the functional impact or disability of work-related upper extremity musculoskeletal pain. Larger, randomized studies are needed to confirm the association.
