# Supplementary material for: Stretching to Reduce Pain-Related Disability Among Echocardiographic and Interventional Laboratory Employees—A Pilot Study
Source: J Soc Cardiovasc Angiogr Interv. 2024 May 2;3(5):101353. doi: 10.1016/j.jscai.2024.101353 (PMC11308027; doi:10.1016/j.jscai.2024.101353)
Supplement: Supplementary Data [file mmc1.pdf]

Survey Research Center

CONSENT

Mayo Clinic: Office for Human Research Protection Consent Form

Stretching exercises and musculoskeletal pain among employees working in the echocardiographic, ultrasound, and interventional laboratories

Principal Investigator: Mandeep Singh, M.D., M.P.H.

You are being asked to participate in a research study about the impact of stretching exercises in reducing work-related pain. We are contacting you because we identified you as an employee working in the radiation/echocardiography/ultrasound suites at Mayo Clinics or Mayo Clinic Health System sites. We would like to invite you to participate since a recent multisite, case-control Mayo Clinic study revealed that high numbers of consultants, RNs, sonographers, and technologists had musculoskeletal pain working in radiology and cardiology departments.

If you agree to participate in stretches, please fill out this survey and the same survey will be sent to you at one year. We will also send you a video demonstration of daily stretches. The stretching routine can be completed in 15 minutes and can be done at any time during the day. We will send you a 2 question survey every 2 weeks to note the number of days that you have done your stretches and to report ant stretch-related injuries.

If you choose not to do stretches, please complete the survey and the same survey will be sent to you in one year.

The risks of this research study are minimal and are not different than what you would experience during your daily life. The risks associated with the research study are time spent during stretching exercises. You will not be paid for time to participate in the study.

Please understand your participation is voluntary and you have the right to withdraw your consent or discontinue participation at any time without penalty.

If you have any questions about this research study, you can contact me at 507-266-3089. If you have any concerns, complaints, or general questions about research or your rights as a participant,

please contact the Mayo Institutional Review Board (IRB) to speak to someone independent of the research team at 507-266-4000 or toll free at 866-273-4681.

- **YES, I agree to participate**

ELIGIBILITY

Do you work in Cath or EP labs, ultrasound, echocardiography laboratories, or in radiology area that requires you to wear lead apron?

- No
- Yes

Are you currently pregnant?

- No
- Yes

STRETCHES

Do you want to participate in doing the stretches for the STRETCH trial?

- **NO**, I choose not to do the daily stretches. Please send me this survey again in one year.
- **YES**, I want to participate in the daily stretches. Please send me the video demonstration of daily stretches and the survey to record my daily stretches.

MUSCULOSKELETAL PAIN

Prior to the start of your profession, did you have any pre-existing musculoskeletal condition(s)?

- No
- Yes

How has your musculoskeletal condition(s) changed since the start of your current profession?

- It has gotten better

- It has stayed the same
- It has gotten worse

How would you describe changes in your musculoskeletal health over the past year?

- It has gotten better
- It has stayed the same
- It has gotten worse

Since starting your current position, have you ever had pain you believe to be work-related?

- No
- Yes

Did any of the following result from your pain?

	No	Yes
I had an ergonomic evaluation of my work environment.	○	○
I was/am on work restriction.	○	○
I missed time at work due to treatment/management of the pain.	○	○
I sought medical care due to the pain.	○	○

How many days of work have you missed in the past 6 months due to pain you believe to be work-related? (Enter 0 if you have not missed days.)

${e://I days

Are you currently bothered by any pain you believe to be work-related?

- No
- Yes

On a scale of 0 to 10, with 0 being "No pain" and 10 being "Worst possible pain," please rate how bad your pain is right now.

- 0 - No pain
- 1
- 2
- 3
- 4
- 5
- 6
- 7
- 8
- 9
- 10 - Worst possible pain

Please mark the ONE descriptor below that best describes your present pain.

- No pain
- Mild
- Discomforting
- Distressing
- Horrible
- Excruciating

During the past 2 weeks, how often have you taken medication (of any kind) to relieve work-related pain?

- Never
- Occasionally, not every day
- Almost every day
- Every day

Do you ever walk or use a bicycle for at least 10 minutes continuously to get to places?

- No
- Yes

In a typical week, how many days do you walk or ride a bicycle for at least 10 minutes continuously to get to places?

${e://I days

How much time do you spend walking or bicycling for travel on a typical day?

${e://I minutes

${e://I hours

Do you do any vigorous-intensity sports, fitness, or recreational (leisure) activities that cause a large increase in breathing or heart rate like running or football for at least 10 minutes continuously?

- No
- Yes

In a typical week, on how many days do you do vigorous–intensity sports, fitness, or recreational (leisure) activities?

${e://I days

How much time do you spend doing vigorous intensity sports, fitness, or recreational activities on a typical day?

${e://I minutes

${e://I hours

Do you do any moderate-intensity sports, fitness, or recreational (leisure) activities that cause small increase in breathing or heart rate such as brisk walking, cycling, swimming, or volleyball for at least 10 minutes continuously?

- No
- Yes

In a typical week, on how many days do you do moderate–intensity sports, fitness, or recreational (leisure) activities?

${e://F days

How much time do you spend doing vigorous intensity sports, fitness, or recreational activities on a typical day?

${e://F minutes

${e://F hours

How much time do you usually spend sitting or reclining on a typical day?

${e://F minutes

${e://F hours

ROLAND-MORRIS DISABILITY QUESTIONNAIRE

As you read the list, think of yourself today. When you read a sentence that describes you today, check the box. If the sentence does not describe you, then leave the box blank and go on to the next one. Remember, only mark the sentence if you are sure it describes you today.

- ☐ I stay at home most of the time because of my back.
- ☐ I change position frequently to try and get my back comfortable.
- ☐ I walk more slowly than usual because of my back.
- ☐ Because of my back, I am not doing any of the jobs that I usually do around the house.
- ☐ Because of my back, I use a handrail to get upstairs.
- ☐ Because of my back, I lie down to rest more often.
- ☐ Because of my back, I have to hold on to something to get out of an easy chair.
- ☐ Because of my back, I try to get other people to do things for me.
- ☐ I get dressed more slowly than usual because of my back.
- ☐ I only stand for short periods of time because of my back.

- ☐ Because of my back, I try not to bend or kneel down.
- ☐ I find it difficult to get out of a chair because of my back.
- ☐ My back is painful almost all the time.
- ☐ I find it difficult to turn over in bed because of my back.
- ☐ My appetite is not very good because of my back pain.
- ☐ I have trouble putting on my socks (or stockings) because of the pain in my back.
- ☐ I only walk short distances because of my back.
- ☐ I sleep less well because of my back.
- ☐ Because of my back pain, I get dressed with help from someone else.
- ☐ I sit down for most of the day because of my back.
- ☐ I avoid heavy jobs around the house because of my back.
- ☐ Because of my back pain, I am more irritable and bad-tempered with people than usual.
- ☐ Because of my back, I go upstairs more slowly than usual.
- ☐ I stay in bed most of the time because of my back.

DASH

Please rate your ability to do the following activities in the last week by marking the appropriate response based on the difficulty. Please answer all questions.

	No difficulty	Mild	Moderate	Severe	Unable to perform
Open a tight or new jar	○	○	○	○	○
Write	○	○	○	○	○
Turn a key	○	○	○	○	○
Prepare a meal	○	○	○	○	○
Push open a heavy door	○	○	○	○	○
Place an object on a shelf above your head	○	○	○	○	○

	No difficulty	Mild	Moderate	Severe	Unable to perform
Do heavy household chores (e.g., wash walls or floors)	○	○	○	○	○
Garden or do yard work	○	○	○	○	○
Make a bed	○	○	○	○	○
Carry a shopping bag or briefcase	○	○	○	○	○

	No difficulty	Mild	Moderate	Severe	Unable to perform
Carry a heavy object (over 10 lbs.)	○	○	○	○	○
Change a lightbulb overhead	○	○	○	○	○

	No difficulty	Mild	Moderate	Severe	Unable to perform
Wash or blow-dry your hair	○	○	○	○	○
Wash your back	○	○	○	○	○
Put on a pullover sweater	○	○	○	○	○
Use a knife to cut food	○	○	○	○	○
Recreational activities which require little effort (e.g., card playing, knitting, etc.)	○	○	○	○	○
Recreational activities in which you take some force or impact through your arm, shoulder, or hand (e.g., golf, hammering, tennis, etc.)	○	○	○	○	○

	No difficulty	Mild	Moderate	Severe	Unable to perform
Recreational activities in which you move your arm freely (e.g., playing frisbee, badminton, etc.)	○	○	○	○	○
Manage transportation needs (getting from one place to another)	○	○	○	○	○
Sexual activities	○	○	○	○	○

During the past week, to what extent an arm, shoulder, or hand problem interfered with your normal social activities with family, friends, neighbors, or groups?

- ○ Not at all
- ○ Slightly
- ○ Moderately
- ○ Quite a bit
- ○ Extremely

During the past week, were you limited in your work or other regular daily activities as a result of an arm, shoulder, or hand problem?

- ○ Not at all
- ○ Slightly
- ○ Moderately

- Quite a bit
- Extremely

Please rate the severity of the following symptoms in the last week.

	None	Mild	Moderate	Severe	Extreme
Arm, shoulder, or hand pain when you performed any specific activity.	○	○	○	○	○
Tingling (pins and needles) in your arm, shoulder, or hand.	○	○	○	○	○
Weakness in your arm, shoulder, or hand.	○	○	○	○	○
Stiffness in your arm, shoulder, or hand.	○	○	○	○	○

During the past week, how much difficulty have you had sleeping because of the pain in an arm, shoulder, or hand?

- No difficulty
- Mild
- Moderate
- Severe
- I can’t sleep from pain

I feel less capable, less confident, or less useful because of an arm, shoulder, or hand problem.

- Strongly disagree
- Disagree
- Neither agree nor disagree
- Agree
- Strongly agree

The following questions ask about the impact of an arm, shoulder, or hand problem on your ability to work. Please mark the answers that best describes your physical ability in the past week.

Did you have any difficulty:

	No	Mild	Moderate	Severe	Unable
Using your usual technique for your work?	○	○	○	○	○
Doing your usual work because of arm, shoulder, or hand pain?	○	○	○	○	○
Doing your work as well as you would like?	○	○	○	○	○
Spending your usual amount of time doing your work?	○	○	○	○	○

NECK DISABILITY INDEX

These questions have been designed to give us information as to how neck pain has affected your ability to manage in everyday life. Please answer every section and mark in each section only the one box that applies to you. We realize you may consider that two or more statements in any one section relate to you, but please just mark the box that most closely describes your problem.

Section 1: Pain Intensity

- ○ I have no pain at the moment.
- ○ The pain is very mild at the moment.
- ○ The pain is moderate at the moment.
- ○ The pain is fairly severe at the moment.
- ○ The pain is very severe at the moment.
- ○ The pain is the worst imaginable at the moment.

Section 2: Personal Care (Washing, Dressing, etc.)

- ○ I can look after myself normally without causing extra pain.
- ○ I can look after myself normally but it causes extra pain.
- ○ It is painful to look after myself and I am slow and careful.
- ○ I need some help but can manage most of my personal care.
- ○ I need help every day in most aspects of self-care.
- ○ I do not get dressed; I wash with difficulty and stay in bed.

Section 3: Lifting

- I can lift heavy weights without extra pain.
- I can lift heavy weights but it gives extra pain.
- Pain prevents me lifting heavy weights off the floor, but I can manage if they are conveniently placed, for example on a table.
- Pain prevents me from lifting heavy weights but I can manage light to medium weights if they are conveniently positioned.
- I can only lift very light weights.
- I cannot lift or carry anything.

Section 4: Reading

- I can read as much as I want to with no pain in my neck.
- I can read as much as I want to with slight pain in my neck.
- I can read as much as I want with moderate pain in my neck.
- I can’t read as much as I want because of moderate pain in my neck.
- I can hardly read at all because of severe pain in my neck.
- I cannot read at all.

Section 5: Headaches

- I have no headaches at all.
- I have slight headaches, which come infrequently.
- I have moderate headaches, which come infrequently.
- I have moderate headaches, which come frequently.
- I have severe headaches, which come frequently.
- I have headaches almost all the time.

Section 6: Concentration

- I can concentrate fully when I want to with no difficulty.
- I can concentrate fully when I want to with slight difficulty.
- I have a fair degree of difficulty in concentrating when I want to.
- I have a lot of difficulty in concentrating when I want to.

- ◯ I have a great deal of difficulty in concentrating when I want to.
- ◯ I cannot concentrate at all.

Section 7: Work

- ◯ I can do as much work as I want to.
- ◯ I can only do my usual work, but no more.
- ◯ I can do most of my usual work, but no more.
- ◯ I cannot do my usual work.
- ◯ I can hardly do any work at all.
- ◯ I can’t do any work at all.

Section 8: Driving

- ◯ I can drive my car without any neck pain.
- ◯ I can drive my car as long as I want with slight pain in my neck.
- ◯ I can drive my car as long as I want with moderate pain in my neck.
- ◯ I can’t drive my car as long as I want because of moderate pain in my neck.
- ◯ I can hardly drive at all because of severe pain in my neck.
- ◯ I can’t drive my car at all.

Section 9: Sleeping

- ◯ I have no trouble sleeping.
- ◯ My sleep is slightly disturbed (less than 1 hr. sleepless).
- ◯ My sleep is mildly disturbed (1-2 hrs. sleepless).
- ◯ My sleep is moderately disturbed (2-3 hrs. sleepless).
- ◯ My sleep is greatly disturbed (3-5 hrs. sleepless).
- ◯ My sleep is completely disturbed (5-7 hrs. sleepless).

Section 10: Recreation

- ◯ I am able to engage in all my recreation activities with no neck pain at all.
- ◯ I am able to engage in all my recreation activities, with some pain in my neck.

- ○ I am able to engage in most, but not all, of my usual recreation activities because of pain in my neck.
- ○ I am able to engage in a few of my usual recreation activities because of pain in my neck.
- ○ I can hardly do any recreation activities because of pain in my neck.
- ○ I can't do any recreation activities at all.

DEMOGRAPHICS

How many years have you been in your current profession?

${e://F years

Which area do you work at the most?

- ○ Catheterization lab
- ○ Echocardiography lab
- ○ Radiology
- ○ Other, please specify: ________

What best describe your job position?

- ○ Catheterization lab nurse
- ○ Catheterization lab tech
- ○ Radiology lab tech
- ○ Radiology lab assistant
- ○ Echocardiography lab nurse
- ○ Echocardiography lab sonographer
- ○ Consultant catheterization lab
- ○ Fellow catheterization lab
- ○ Consultant radiology
- ○ Consultant echocardiographic lab
- ○ Fellow echocardiographic lab
- ○ Other, please specify: ________

Do you perform/assist with procedures that expose you to radiation?

- No
- Yes

How much time , on average, do you spend performing/assisting with these procedures per week?

${e://I minutes

${e://I hours

How much time, on average, do you spend wearing a lead apron per week?

${e://I minutes

${e://I hours

How many years have you been involved in procedures that exposure you to radiation?

${e://I years

What type of lead apron do you wear?

- Single
- Two-piece

During these procedures, how often do you...

	Never	Occasionally	Most of the time	All of the time
Wear lead eye protection?	○	○	○	○
Remove your lead apron?	○	○	○	○
Wear soft-soled or athletic shoes?	○	○	○	○
Perform specific stretching/exercises before and/or after the procedure?	○	○	○	○

	Never	Occasionally	Most of the time	All of the time
Use a glass shield from the ceiling and on the floor?	◯	◯	◯	◯

END/SUBMIT

How old are you?

◯ Less than 20 years
◯ 20 to 30 years
◯ 31 to 40 years
◯ 41 to 50 years
◯ 51 to 60 years
◯ 61 to 70 years
◯ More than 70 years

How do you describe yourself?
(Mark all that apply.)

☐ Female
☐ Male
☐ Transgender
☐ Do not identify as female, male, or transgender

What is your weight? (Please round to nearest whole number.)

${e://I pounds

What is your height? (Please round to nearest whole number.)

${e://I feet

${e://I inches

THANK YOU FOR COMPLETING THE SURVEY!

Please click SUBMIT to record your answers.
